# Differences Between Female Singers With Phonotrauma and Vocally Healthy Matched Controls in Singing and Speaking Voice Use During 1 Week of Ambulatory Monitoring

**DOI:** 10.1044/2020_AJSLP-20-00227

**Published:** 2021-01-20

**Authors:** Laura E. Toles, Andrew J. Ortiz, Katherine L. Marks, James A. Burns, Tiffiny Hron, Jarrad H. Van Stan, Daryush D. Mehta, Robert E. Hillman

**Affiliations:** a Massachusetts General Hospital, Boston; b MGH Institute of Health Professions, Boston, MA; c Harvard Medical School, Boston, MA

## Abstract

**Purpose:**

Previous ambulatory voice monitoring studies have included many singers and have combined speech and singing in the analyses. This study applied a singing classifier to the ambulatory recordings of singers with phonotrauma and healthy controls to determine if analyzing speech and singing separately would reveal voice use differences that could provide new insights into the etiology and pathophysiology of phonotrauma in this at-risk population.

**Method:**

Forty-two female singers with phonotrauma (vocal fold nodules or polyps) and 42 healthy matched controls were monitored using an ambulatory voice monitor. Weeklong statistics (average, standard deviation, skewness, kurtosis) for sound pressure level (SPL), fundamental frequency, cepstral peak prominence, the magnitude ratio of the first two harmonics (*H_1_–H_2_*), and three vocal dose measures were computed from the neck surface acceleration signal and separated into singing and speech using a singing classifier.

**Results:**

Mixed analysis of variance models found expected differences between singing and speech in each voice parameter, except SPL kurtosis. SPL skewness, SPL kurtosis, and all *H_1_–H_2_* distributional parameters differentiated patients and controls when singing and speech were combined. Interaction effects were found in *H_1_–H_2_* kurtosis and all vocal dose measures. Patients had significantly higher vocal doses in speech compared to controls.

**Conclusions:**

Consistent with prior work, the pathophysiology of phonotrauma in singers is characterized by more abrupt/complete glottal closure (decreased mean and variation for *H_1_–H_2_*) and increased laryngeal forces (negatively skewed SPL distribution) during phonation. Application of a singing classifier to weeklong data revealed that singers with phonotrauma spent more time speaking on a weekly basis, but not more time singing, compared to controls. Results are used as a basis for hypothesizing about the role of speaking voice in the etiology of phonotraumatic vocal hyperfunction in singers.

The development of benign vocal fold lesions such as nodules or polyps due to phonotraumatic vocal hyperfunction (PVH) represents some of the most common types of voice disorders (Bhattacharyya, 2014). There is relative consensus that PVH is associated with cumulative vocal fold tissue damage and/or reaction to continuous tissue inflammation that might be perpetuated by engaging in daily vocal behaviors such as phonating too loudly without adequate rest periods, using inappropriate pitch, or recruiting inefficient phonation patterns (Mehta et al., 2012; Popolo et al., 2005). Thus, individuals in professions that require high voice use (e.g., teachers and call center professionals) have a higher incidence and risk of developing phonotraumatic voice disorders (Cantarella et al., 2014; Roy et al., 2004).

Professional singers represent a substantial portion of patients who present to voice clinics. While singers represent less than 1% of the workforce in the United States, studies have estimated that they comprise 11.5%–29% of treatment-seeking individuals in voice clinics, which is a substantial disproportion (Kridgen, 2019; Titze et al., 1997). This high rate of treatment seeking might be attributed to singers' complex vocal needs (e.g., sustained phonation throughout a wide range of pitch and intensity), leading to increased incidence of voice disorders, as well as the devastating impact a voice disorder could have on a singer's career (Pestana et al., 2017; Phyland, 2017; Phyland et al., 1999). Like other high voice use occupations, singers are more likely to develop phonotrauma than other types of voice disorders (Roy et al., 2005; Titze et al., 1997; Verdolini & Ramig, 2001). The higher vocal demand of singing is commonly assumed to be the primary factor that leads to phonotrauma in some singers, but this does not explain why other singers with similar singing-related vocal demands do not develop phonotrauma. This observation suggests that there may be additional factors that contribute to phonotrauma in singers that are not solely related to the higher vocal demands of singing.

One sparsely addressed area in the literature is the impact the speaking voice might have on a singer's vocal health. Many professional, semiprofessional, and student singers often have additional vocational responsibilities to make ends meet, such as jobs in the service industry that might entail frequent use of speaking voice. Additionally, a social component involving speaking in large groups over background noise could accompany voice performances. Though singers are often counseled to practice voice conservation (limit voice use outside singing practice and performance), the actual relative contributions of singing and speaking voice use to the development of phonotrauma in singers is unknown.

Ambulatory voice monitoring using a neck-placed sensor, often a miniature accelerometer (ACC), has the ability to provide objective characterization of voice use during daily life (Cheyne et al., 2003; Popolo et al., 2005; Searl & Dietsch, 2014; Szabo et al., 2001). Studies employing ambulatory voice monitoring technology have traditionally measured fundamental frequency (*f*_0_), vocal intensity, and vocal dose (estimates of vocal load) as averages, standard deviations, and total accumulations to describe vocal behavior and/or to indirectly estimate exposure of vocal fold tissue to the mechanical stress that occurs during phonation (Carroll et al., 2006; Hunter & Titze, 2009; Schloneger & Hunter, 2017). Many of these studies have been conducted to identify occupational safety standards in terms of vocal fold vibration exposure within cohorts of individuals with healthy voices that have higher-than-average occupational voice use requirements (Cantarella et al., 2014; Hunter & Titze, 2010; Schloneger & Hunter, 2017; Titze et al., 2007).

In recent years, several studies have used ACC-based weeklong ambulatory voice monitoring to determine if there are differences in voice use between patients with PVH and matched controls (Cortés et al., 2018; Ghassemi et al., 2014; Mehta et al., 2015; Szabo Portela et al., 2018; Van Stan et al., 2020, 2015). Somewhat surprisingly, all of these studies found no statistically significant differences in average measures of vocal intensity, *f*_0_, or vocal doses (i.e., amount of voice use) between patients with PVH and matched controls, which seems at odds with clinical assumptions about the role of voice use in PVH. However, a recent study (Van Stan et al., 2020) added an examination of distributional characteristics of ambulatory voice use and measures indicative of glottal closure. Results showed that the weekly voice use of patients with PVH reflected higher sound pressure level (SPL) tendencies (negatively skewed SPL) with more abrupt/complete glottal closure (reduced first and second harmonics [*H_1_–H_2_*] variability, especially toward higher values of frame-based *H_1_–H_2_*) and reduced *f*_0_ variability. Moreover, the results of a logistic regression showed that a combination of SPL skewness and *H_1_–H_2_* variability could classify patients and controls based on their weekly voice data, with an area under the receiver operating characteristic curve of .85 and .82 on training and test sets, respectively.

Singers made up a large percentage (70%–100%) of participants in a majority of the ambulatory voice monitoring studies of PVH that have been conducted (Cortés et al., 2018; Ghassemi et al., 2014; Mehta et al., 2015; Van Stan et al., 2020, 2015). In all of these studies, measures of voice use are based on combining data from all types of phonation, including speech and singing. It is not known whether combining data from speech and singing obscures potentially salient differences in voice use between patients and controls that might have otherwise been seen if singing and speech would have been analyzed separately. Doing so is particularly critical if the goal is to determine the relative contribution of speech and singing to PVH in singers.

Until recently, no objective tool existed for disaggregating singing from speech in recordings of the ambulatory ACC signal. Previous studies investigating vocal dose and vocal function measures in singing and speech in healthy singers have relied on subject-generated activity logs to identify time blocks during their day when they reported that they were generally engaged in singing or speaking (Gaskill et al., 2013; Schloneger et al., 2011; Schloneger & Hunter, 2017). To avoid reliance on subjective reports of voice activity, we have recently developed an automatic singing and speech classification method for the ACC signal. The initial validation of this method showed high accuracy in detecting singing and speech on both a training set and separate test set of patients with PVH and controls with healthy voices (Ortiz et al., 2019).

Using this objective singing classifier, our group recently conducted a study investigating weeklong ambulatory voice recordings among a large cohort (*n* = 64) of vocally healthy female college student singers and found (not surprisingly) that speaking and singing voice characteristics were significantly different when the two types of phonation were separated (Toles et al., 2020). Female college student singers were found to have only phonated for 8.4% of the total time monitored, 26% of which was spent singing and 74% of which was spent speaking. Even though the singing voice could be considered the “occupational voice” among this group, student singers used their voices for speaking 3 times more than for singing during a typical school week. There were also several notable differences in voice use parameters between singing and speech. Among the expected differences, we found that average SPL was higher in singing than in speech by 3.0 dB, *f*_0_ mode was higher in singing than speech by 121.9 Hz, and singing had more *f*_0_ variability than speech—all of which reflects the greater vocal demands of singing. We also found that cepstral peak prominence (CPP) was significantly higher, and *H_1_–H_2_* values were significantly lower in speech than singing. These differences were interpreted as indicating that everyday speech is produced with more abrupt and/or complete glottal closure (lower *H_1_–H_2_* values), with resultant higher average levels of periodic energy (higher CPP values) than singing—all of which may be at least partially attributed to the impact on vocal fold vibratory kinematics of the higher *f*_0_ modes associated with singing. More abrupt/complete glottal closure is assumed to reflect increased potential for phonotrauma. While it is universally acknowledged that the act of singing is typically more vocally demanding than everyday speaking, the findings in this study suggest that speaking has the potential to play a larger role in perpetuating phonotrauma in singers than is reflected in current views that place the onus on singing voice use, especially when accompanied by the fact that the students used their voices for speaking 3 times more than for singing.

The purpose of the current investigation is to determine whether separating speech and singing in the analysis of ambulatory voice recordings will better differentiate between singers with phonotrauma and singers with normal voices in terms of measures that reflect daily voice use. We hypothesize that separating singing from speech will reveal differences between these two groups that have not been seen in previous studies that have combined these two types of phonation. The results of this investigation have the potential to provide insights into the relative contributions of the singing versus the speaking voice to the etiology and pathophysiology of phonotrauma in singers.

## Method

### Participants

Forty-two female patients with a diagnosis of bilateral vocal fold nodules (*n* = 39) or unilateral vocal fold polyp (*n* = 3) who self-identified as professional, semiprofessional, or student singers were recruited through sequential convenience sampling at the Massachusetts General Hospital Voice Center. Only female participants were selected to be in this study in order to provide a homogenous sample of a group that has a significantly higher incidence of phonotraumatic vocal fold lesions. Diagnoses were based on a comprehensive team evaluation (laryngologist and speech-language pathologist [SLP]) at the Massachusetts General Hospital Voice Center that included (a) the collection of a complete case history, (b) stroboscopic imaging of the larynx, (c) an auditory-perceptual evaluation using the Consensus Auditory-Perceptual Evaluation of Voice (Kempster et al., 2009), and (d) aerodynamic and acoustic assessment of vocal function. Table 1 presents patients' scores for the Consensus Auditory-Perceptual Evaluation of Voice (*n* = 42) and the self-rated Voice-Related Quality of Life (*n* = 40; Hogikyan & Sethuraman, 1999). Forty-two control subjects who also self-identified as singers with no histories of voice disorders were each matched to a patient according to approximate age (±5 years), sex, occupation, and style of singing (classical or nonclassical). The normal vocal status of all control participants was verified via interview with a licensed SLP and a laryngeal stroboscopic examination. Potential control subjects were excluded from the study if they (a) indicated present or past vocal difficulties, (b) participated in previous voice therapy or laryngeal surgery, (c) demonstrated an abnormal voice quality during an auditory-perceptual evaluation by the SLP, or (d) demonstrated structural abnormalities on the laryngeal stroboscopic examination.

**Table 1.** Patients' self-reported impact on quality of life due to their voice disorder using the Voice-Related Quality of Life (V-RQOL) subscales and the perceived voice quality judged by a speech-language pathologist using the Consensus Auditory-Perceptual Evaluation of Voice (CAPE-V) form.

Measure	*M* (*SD*)
CAPE-V	
Overall Severity	22.8 (11.9)
Roughness	16.2 (12.9)
Breathiness	12.5 (12.9)
Strain	18.0 (9.9)
Pitch	4.2 (7.9)
Loudness	4.5 (9.2)
V-RQOL	
Social–Emotional	68.5 (23.0)
Physical Functioning	73.3 (18.3)
Total score	71.5 (17.8)

*Note.* Mean and standard deviation reported for each subscale measure. The CAPE-V was administered for the total patient cohort (*n* = 42). V-RQOL scores were available for 40 patients.

The average age (standard deviation) of singers with phonotrauma and matched control singers was 23.0 (8.3) and 22.9 (6.6) years, respectively. Six (14%) of the patient–control matched pairs were composed of professional singers, four of which were classical singers and two were nonclassical singers. Thirty-six (86%) of the matched pairs were composed of college students enrolled in a voice-related music program, eight of which were classical singers and 28 were nonclassical singers.

### Data Collection

Ambulatory voice monitoring data were acquired using the custom-designed voice health monitor (VHM) that has been previously described in detail (Mehta et al., 2015, 2012). To summarize, the VHM system consists of a miniature ACC (model BU-27135, Knowles Electronics) positioned on the subglottal neck surface paired to a custom smartphone application. The system records the unprocessed ACC signal at an 11,025-Hz sampling rate, 16-bit quantization, and 80-dB dynamic range and obtains frequency content of neck surface vibrations up to 5000 Hz.

Participants in the patient group were monitored for 7 days before any surgical and/or therapeutic intervention for the phonotraumatic lesions. Each enrolled matched control participant was also monitored for one full week. All participants were monitored during weeks that involved typical vocal demands (e.g., student singers were monitored during a week in which school was in session) and were instructed to participate in typical voice use activities while being monitored (e.g., rehearsals, performances, social activities). Each morning, the VHM application prompted the participant to complete a daily SPL calibration sequence, which was recorded by a small hand-held microphone (H1 Handy Recorder, Zoom Corporation) positioned 15 cm from the lips (Mehta et al., 2012; Švec et al., 2005). These daily calibrations allowed for calculations of ACC-based estimates of SPL and provided ongoing verification that the system was operating properly.

### Voice Feature Extraction

Ambulatory weeklong voice monitoring data were processed to yield voice features from the raw ACC signal. Previous publications have described the methods used to calibrate the ACC signal to SPL and to distinguish between voiced and nonvoiced activities (i.e., voice activity detection) in the ACC signal, which were used in this study (Mehta et al., 2015, 2012). To summarize, measures of *f*_0_ and SPL were extracted from nonoverlapping 50-ms frames. Acoustic SPL measures were estimated from the ACC signal through the daily calibration routine that records ACC and acoustic signals simultaneously. Voiced activity was distinguished from nonvoiced activity if the frames met the following thresholds: (a) SPL greater than 45 dB SPL at 15 cm, (b) *f*_0_ between 70 and 1000 Hz, (c) the first nonzero lag peak in the normalized autocorrelation greater than .6, and (d) the ratio of low- to high-frequency energy exceeding 22 dB.

CPP was extracted from the raw ACC signal using two discrete Fourier transforms computed in succession with a logarithmic transformation between them. We defined CPP as the difference between the magnitude of the highest peak and the baseline regression level in the power cepstrum. The peak search was limited to frequencies between 2.4 and 12.0 ms, which corresponds to frequencies of 416.7 and 83.3 Hz, respectively (Mehta et al., 2016, 2015). We derived *H_1_–H_2_* from a 1,024-point fast Fourier transform of each 50-ms voiced frame. The measure represents the difference between magnitudes (dB) of the first and second harmonics in the frequency spectrum (Mehta et al., 2019).

Three average vocal dose measures were also computed for each participant (Titze et al., 2003). Time dose (accumulated phonation time) was calculated and normalized as the percentage of phonation during the total time the participant was being monitored. Cycle dose was computed as the number of vocal fold oscillations during the total monitoring time, normalized based on hours monitored (cycles per hour). Distance dose was calculated as the cumulative distance traveled by the vocal folds during the total monitoring time. This calculation incorporates estimates of cycle dose with estimates of vibratory amplitude based on SPL (Titze et al., 2003; Van Stan et al., 2015), which was then normalized by hours monitored (meters per hour).

### Singing Detection

Further processing of the ACC-based data included application of an automated singing classifier to separate singing from speech per frame, allowing for each phonation mode to be analyzed independently. Detailed description of the classification process that was applied to the current data can be found in a recent publication in which the singing classifier performed with an overall accuracy of 93.3%, sensitivity of 90.3%, and specificity of 96.4% on a training set. Overall accuracy improved to 94.2% on a held-out test set (Ortiz et al., 2019). Following the application of the singing detector, all of the measures of vocal function and dose were calculated for singing and speech separately for each participant.

Since an ongoing focus of our group is to develop new and improved methods for extraction of phonatory measures from the neck surface ACC signal, we completed a quality check to assess the performance of the singing classifier on the data for this study (screening for misclassifications). Distributions for the two input features of the classifier, the normalized autocorrelation peak and *f*_0_ modes, were examined for each subject week. Subject weeks that showed a lack of clear separation between the labeled “singing” and “speech” distributions were reviewed by a trained listener. When this process started, there were 60 matched pairs of patients and control participants. Fifteen singers with phonotrauma and 17 control participants were determined to have misclassification rates that exceeded the specifications of the classifier. When the original pairing of participants was considered (i.e., the entire pair was eliminated if one member of the pair was eliminated), 36 pairs were left. This reduction was partially offset by creating new pairings for six patients and six controls based on the original matching criteria, which resulted in a total of 42 pairs that could be analyzed.

### Statistical Analysis

Within-subject summary statistics were computed for the weeklong distributions of SPL, *f*_0_, CPP, and *H_1_–H_2_*, which included the distributional parameters of mean (mode for *f*_0_), standard deviation, skewness, and kurtosis, computed separately for singing and speech*.* We also calculated phonation time (%), cycle dose (cycles per hour), and distance dose (meters per hour) for each type of phonation. Each of these voice feature parameters are included as dependent variables in our analyses.

To address our hypotheses, we used a 2 × 2 mixed analysis of variance (ANOVA) to evaluate the main effects of diagnosis (patient or control) and type of phonation (singing or speech) and their interaction for each of the voice feature parameters. Diagnosis was treated as a between-subjects variable, and type of phonation was treated as a repeated-measures variable. We considered three choices for random effects: matched pair, subject, and subject nested within pair. Comparison of these choices using Akaike's information criterion indicated that a single random effect for “subject” best fit the data. We note that, since there are only two values per subject per phonation mode, the model assumptions and resulting significance values for this ANOVA model are equivalent to those corresponding to a mixed-model regression. We felt that ANOVA best represented our research questions, which would allow for straightforward interpretation of the analysis compared to the mixed-model regression. To account for the multiple ANOVA models being conducted, we corrected the alpha level of significance using a familywise error correction (alpha of .05 divided by the number of tests conducted within a variable family). Each dependent variable was a member of one of five families of features (SPL, *f*_0_, CPP, *H_1_–H_2_*, and dose). Four ANOVA models (mean/mode, standard deviation, skewness, and kurtosis) were conducted for the families of *f*_0_, SPL, CPP, and *H_1_–H_2_*, so the alpha significance value was divided by 4 (α = .0125). Three models were conducted in the vocal dose family of measures (phonation time, cycle dose, and distance dose), so the alpha was divided by 3 (α = .0166).

For voice parameters that showed a significant interaction effect (Diagnosis × Phonation Mode), we conducted paired *t* tests to assess differences between the summary statistics of weekly voice use for each type of phonation. The alpha level of significance was also corrected for paired *t* tests using the familywise error correction, as detailed above. When statistical significance was found, a Cohen's *d* effect size (the difference between the means of the two groups divided by the standard deviation of the difference) was calculated to better characterize the magnitude of the differences between groups. Effect sizes were interpreted according to Cohen's standardized method (small, ≤ .19; small-to-medium, .20–.49; medium-to-large, .50–.79; large, ≥ .80; Cohen, 1988).

## Results

The results obtained for the main effects of phonation type (singing compared to speech) using the 2 × 2 mixed ANOVA models are shown in Table 2. All of the main effects for type of phonation were significant, except SPL kurtosis. Participants spent significantly more time speaking than singing (higher vocal doses for speaking), and singing was produced with higher average values and more variation of SPL and *f*_0_, lower mean and variation for CPP, and higher mean and variation for *H_1_–H_2_*.

**Table 2.** Main effects of phonation type (singing or speech) are presented with means (standard deviations) of the weekly summary statistics of sound pressure level (SPL), fundamental frequency (*f*_0_), cepstral peak prominence (CPP), the difference between the first two harmonics (*H_1_–H_2_*), and vocal dose measures for combined participant groups (patients and controls) in singing and speech separately.

Variable	Main effect *p*	Cohen's *d*	Singing *M* (*SD*)	Speech *M* (*SD*)
SPL				
*M* (dB SPL)	< .0001*	0.80	88.78 (5.99)	84.43 (4.76)
*SD* (dB)	< .0001*	0.32	12.47 (2.63)	11.70 (2.12)
Skewness	< .0001*	0.49	−0.31 (0.37)	−0.15 (0.28)
Kurtosis	.1196	—	3.23 (0.70)	3.12 (0.35)
*f*_0_				
Mode (Hz)	< .0001*	2.27	319.86 (42.95)	199.62 (19.17)
*SD* (Hz)	< .0001*	2.91	95.36 (14.24)	61.23 (8.54)
Skewness	< .0001*	2.84	1.12 (0.35)	2.25 (0.44)
Kurtosis	< .0001*	2.49	5.16 (1.60)	12.30 (3.73)
CPP				
*M* (dB)	< .0001*	1.00	21.82 (1.51)	23.15 (1.12)
*SD* (dB)	< .0001*	1.56	3.97 (0.36)	4.51 (0.33)
Skewness	< .0001*	1.15	−0.03 (0.30)	−0.31 (0.17)
Kurtosis	< .0001*	0.79	2.60 (0.30)	2.41 (0.16)
*H_1_–H_2_*				
*M* (dB)	< .0001*	2.34	9.05 (2.33)	4.03 (1.95)
*SD* (dB)	< .0001*	1.29	7.31 (0.81)	6.29 (0.77)
Skewness	.0001*	0.24	0.55 (0.25)	0.69 (0.77)
Kurtosis	< .0001*	1.42	3.07 (0.52)	3.97 (0.73)
Dose				
Phonation time (%)	< .0001*	2.91	2.32 (1.19)	7.14 (2.02)
Cycle dose (cycles/hr)	< .0001*	1.70	31,002 (16,950)	59,442 (16,420)
Distance dose (m/hr)	< .0001*	1.59	118.58 (67.50)	240.48 (85.20)

* Significance at or below corrected α value (SPL, *f*_0_, CPP, and *H_1_–H_2_* α = .0125; Dose α = .0166).

Results obtained for the main effects of diagnosis and interaction effects for each of the 2 × 2 mixed ANOVA models are reported in Table 3. Table 4 shows the results of post hoc paired *t* tests that were performed for variables that demonstrated significant interaction effects. Shown are the weeklong statistics for the patients and matched controls for each type of phonation.

**Table 3.** Interactions and main effects of diagnosis (patient or control) are presented with group-based means (standard deviations) of the weekly summary statistics of sound pressure level (SPL), fundamental frequency (*f*_0_), cepstral peak prominence (CPP), the difference between the first two harmonics (*H_1_–H_2_*), and vocal dose measures for combined phonation type (singing or speech).

Variable	Interaction *p*	Diagnosis main effect *p*	Cohen's *d*	Patient *M* (*SD*)	Control *M* (*SD*)
SPL					
*M* (dB SPL)	.1349	.0695	—	86.12 (4.23)	85.03 (5.07)
*SD* (dB)	.1034	.0723	—	11.69 (1.96)	12.61 (2.49)
Skewness	.1447	< .0001*	0.87	−0.26 (0.24)	−0.04 (0.25)
Kurtosis	.0498	.0020*	0.60	3.19 (0.34)	2.94 (0.34)
*f*_0_					
Mode (Hz)	.0363	.0786	—	201.34 (20.41)	202.67 (17.95)
*SD* (Hz)	.6692	.0212	—	82.94 (12.62)	98.04 (18.07)
Skewness	.0666	.3359	—	1.84 (0.45)	1.51 (0.43)
Kurtosis	.4127	.6110	—	8.22 (3.22)	6.15 (2.11)
CPP					
*M* (dB)	.0618	.0193	—	23.15 (0.99)	22.52 (1.26)
*SD* (dB)	.5197	.1400	—	4.48 (0.26)	4.38 (0.37)
Skewness	.1077	.0253	—	−0.27 (0.16)	−0.17 (0.18)
Kurtosis	.6150	.8223	—	2.41 (0.14)	2.38 (0.18)
*H_1_–H_2_*					
*M* (dB)	.0222	.0001*	0.49	4.40 (1.69)	6.19 (1.98)
*SD* (dB)	.0546	.0009*	0.52	6.45 (0.78)	7.34 (0.95)
Skewness	.4764	.0018*	0.49	0.77 (0.24)	0.62 (0.21)
Kurtosis	.0013*	< .0001*	0.71	4.15 (0.68)	3.38 (0.57)
Dose					
Phonation time (%)	< .0001*	.0523	—	9.92 (2.31)	8.93 (2.55)
Cycle dose (cycles/hr)	< .0001*	.6400	—	90,745 (20,261)	89,083 (28,634)
Distance dose (m/hr)	< .0001*	.3205	—	370.46 (103.84)	345.71 (134.28)

*Note.* Means and standard deviations are presented for each group with singing and speech combined.

* Significance at or below corrected α value (*f*_0_, SPL, CPP, and *H_1_–H_2_* α = .0125; Dose α = .0166).

**Table 4.** Group-based means (standard deviations), significance values, and effect sizes of weekly summary statistics for the voice parameters that showed significant interaction effects (diagnosis by phonation type) during the mixed analysis of variance, presented separately for each phonation type (singing and speech).

Variable	Speech				Singing			
	Patient *M* (*SD*)	Control *M* (*SD*)	*p*	Cohen's *d*	Patient *M* (*SD*)	Control *M* (*SD*)	*p*	Cohen's *d*
*H_1_–H_2_*								
Kurtosis	4.35 (0.73)	3.58 (0.49)	< .0001*	0.88	3.20 (0.55)	2.94 (0.45)	.0252	—
Dose								
Phonation time (%)	7.96 (1.94)	6.32 (1.77)	< .0001*	0.67	2.02 (0.91)	2.63 (1.77)	.0262	—
Cycle dose (cycles/hr)	65,620 (14,632)	53,264 (15,922)	.0002*	0.61	26,073 (11,994)	37,930 (19,697)	.0098	—
Distance dose (m/hr)	268.30 (75.31)	212.67 (86.25)	.0010*	0.54	103.73 (51.30)	133.43 (78.35)	.0498	—

* Significance at or below corrected α value (*H_1_–H_2_* α = .025; Dose α = .0083).

### SPL

The results for the mixed ANOVA showed a significant diagnosis main effect with a large effect size for SPL skewness, *F*(1, 82) = 21.6, *p* < .0001, *d* = 0.87, with patients showing a more negatively skewed SPL distribution compared to controls. This indicates that the SPL distribution in combined phonation is skewed to the higher part of the distribution for patients, whereas controls show a more symmetric SPL distribution. The main effect for SPL kurtosis was also significant, *F*(1, 82) = 10.2, *p* = .002, *d* = 0.60, representing a medium-to-large effect size. Although both groups have high SPL kurtosis values in combined phonation, patients' mean SPL kurtosis value is significantly higher than controls, indicating that the majority of SPL variation for patients occurs within a more restricted range compared with controls.

There were no significant interaction effects in any of the SPL distributional parameters, suggesting that the group differences found were not significantly influenced by the type of phonation. Therefore, paired *t* tests were not conducted on any SPL parameters.

### H_1_–H_2_

All four distributional parameters for *H_1_–H_2_* were found to have significant diagnosis main effects. *H_1_–H_2_* mean had a significant diagnosis main effect with a small-to-medium effect size, *F*(1, 82) = 18.2, *p* = .0001, *d* = 0.49. *H_1_–H_2_* was 1.79 dB lower in patients compared to controls. *H_1_–H_2_* standard deviation had a significant diagnosis main effect with a medium-to-large effect size, *F*(1, 82) = 12.0, *p* = .0009, *d* = 0.52, with patients having less variability around the mean than controls. There was also a significant diagnosis main effect of *H_1_–H_2_* skewness with a small-to-medium effect size, *F*(1, 82) = 10.4, *p* = .0018, *d* = 0.49. Patients had a more positively skewed *H_1_–H_2_* distribution than controls for combined phonation, indicating that patients produced more phonation in the lower part of their distributions than controls.

The fourth parameter, *H_1_–H_2_* kurtosis, had a significant diagnosis main effect with a medium-to-large effect size, *F*(1, 82) = 28.7, *p* < .0001, *d* = 0.71, and a significant interaction effect, *F*(1, 82) = 11.0, *p* = .0013. Post hoc paired *t* tests showed a significant difference between groups in *H_1_–H_2_* kurtosis, with a medium-to-large effect size in speech, *t*(41) = 5.7, *p* < .0001, *d* = 0.67. The *H_1_–H_2_* kurtosis value was significantly higher in patients than in controls, indicating that the majority of *H_1_–H_2_* variation for patients occurs within a more restricted range compared with controls.

### f _0_ and CPP

No significant diagnosis main effects or interaction effects were found for *f*_0_ or CPP parameters.

### Vocal Doses

All three vocal dose measures had significant interaction effects but did not show significant main effects for diagnosis. Post hoc paired *t* tests found that the significant interactions were solely related to speech. Phonation time (%) was significantly different between groups in speech with a medium-to-large effect size, *t*(41) = 4.4, *p* < .0001, *d* = 0.67. Patients were found to speak for 7.96% of the total monitoring time, whereas matched controls spoke for 6.32% of the total monitoring time. Phonation time was higher in singing for controls, though this difference only approached the corrected significance value.

Cycle dose values (cycles per hour) were significantly different between groups in speech, *t*(41) = 4.0, *p* = .0002, *d* = 0.61, representing a medium-to-large effect size. Patients had an average of 12,356 more cycles per hour than controls during speech. The difference between groups in singing (controls > patients) also approached but did not meet the corrected significance value.

A similar pattern was found with distance dose values. Distance dose in speaking was 55.63 m/hr greater in patients than controls, *t*(41) = 3.5, *p* = .0010, *d* = 0.54, representing a medium effect size. No significant difference was found in singing.

## Discussion

This study applied a singing classifier to the ambulatory ACC recordings of singers with phonotrauma and healthy controls to determine if analyzing singing and speech separately would reveal differences in voice use that could provide new insights into the etiology and pathophysiology of phonotrauma in this at-risk population. We hypothesized that the separate analysis of singing and speech would reveal additional differences between these two groups that have not been observed in previous studies that included large cohorts of singers and analyzed these two types of phonation together.

A benefit of using a mixed ANOVA on these data is that we were able to analyze the main effects of phonation type (singing vs. speech) and diagnosis (patients vs. controls) as well as interaction effects to determine if differences between and controls are dependent on the type of phonation. Findings for the main effects largely replicated results from previous studies. First, with respect to phonation type, results showing that singers spend significantly more time speaking than singing in a typical week and that singing is produced with higher and more variable levels of SPL, *f*_0_, and *H_1_–H_2_*, completely corroborates results from a previous ambulatory monitoring study of vocally healthy student singers (Toles et al., 2020). Findings in the present investigation of lower and less variable values for CPP in singing are also in agreement with this previous work. Toles et al. (2020) noted that the results for SPL and *f*_0_ were expected based on obvious perceptual differences between singing and speech and that increased levels and variability of these parameters is indicative of the higher demands that singing places on the vocal system. However, the authors went on to suggest that the somewhat unexpected findings for *H_1_–H_2_* and CPP indicate that the more frequent use of speaking voice also has the potential to contribute to phonotrauma because it is produced with more abrupt glottal closure as indicated by lower and less variable *H_1_–H_2_* values (Hillenbrand & Houde, 1996; Klatt & Klatt, 1990; Lowell et al., 2012) and higher magnitudes of periodic energy (indicated by higher CPP values) compared to singing.

Results for the main effects of diagnosis showed that average measures of SPL, *f*_0_, and vocal doses were not significantly different between groups, which is consistent with results from previous studies that included large cohorts of singers and combined speech and singing in the analyses (Mehta et al., 2015; Van Stan et al., 2015). Recent results for higher order distributional characteristics (Van Stan et al., 2020) were also replicated by findings showing a large effect size for the diagnosis main effect of SPL skewness (*d* = 0.87) and medium-to-large effect sizes for the measures of *H_1_–H_2_* variability in terms of the standard deviation (*d* = 0.52) and kurtosis (*d* = 0.71).

Compared to healthy controls, the weekly voice use of singers with PVH was again characterized by the tendency to use higher SPL levels as indicated by a more negatively skewed distribution (Van Stan et al., 2020). Van Stan et al. (2020) acknowledged that “there is some uncertainty about the extent to which negative skewing of the ACC-based SPL distribution reflects comparable increases in the oral SPL (i.e., more frequent use of “louder” speech)” because of limitations in current procedures for SPL calibration of the ACC signal (Bottalico et al., 2018; Švec et al., 2005; Umatani et al., 2020). However, these authors also demonstrated that the SPL skewness correlated well with the skewness of the non-SPL calibrated neck skin acceleration magnitude (physical vibration units of cm/s^2^, in units of dB) which serves to corroborate the significant differences in skewness between patients and controls. It has also been shown that the amplitude of the ACC signal actually correlates better with subglottal pressure than with SPL (Fryd et al., 2016; Marks et al., 2019). Thus, irrespective of the extent to which greater SPL skewness reflects the use of “louder” speech, this finding supports the view that patients with phonotrauma are employing higher laryngeal forces (including subglottal pressure) to phonate than healthy controls (Van Stan et al., 2020).

In addition to replicating the previously reported finding of reduced *H_1_–H_2_* variability for patients with phonotrauma (smaller standard deviation and larger kurtosis indicating less variation toward higher values; Van Stan et al., 2020), this study found that singers with phonotrauma also had significantly lower average *H_1_–H_2_* values than healthy controls for singing and speech combined. Findings also showed that the restriction in *H_1_–H_2_* variability for singers is more associated with speaking than singing voice use (post hoc testing showing significantly larger kurtosis for speech). The finding of lower average *H_1_–H_2_* values that vary less reflects the prevalence of more abrupt/complete glottal closure (Klatt & Klatt, 1990; Mehta et al., 2019; Stephens, 1998) in singers with PVH, which is even more clearly the case during speaking voice use. As proposed by Van Stan et al. (2020), the persistence of more abrupt/complete glottal closure in the presence of vocal fold lesions that can obstruct closure may indicate compensatory hyperadduction (hyperfunction) that is part of the pathogenic “vicious cycle” that has been associated with PVH (Hillman et al., 1989, 2020).

Evidence of compensation for the presence of vocal fold lesions was also indicated by a lack of significant differences between patients and controls for the main effects of measures that reflect voice quality (CPP distributional parameters) and pitch (*f*_0_ distributional parameters). CPP, which correlates with the perception of overall dysphonia (dysphonic voices tend to have lower CPP values compared to healthy voices; Awan et al., 2010), actually trended higher for the patient group (though this difference only approached our corrected significance value, *p* = .0193). The finding of no significant differences for *f*_0_ distributional parameters is partially at odds with previous studies that included singers and nonsingers and found that patients had significantly less *f*_0_ variability (Mehta et al., 2015; Van Stan et al., 2020, 2015). One interpretation of the current findings is that compensatory mechanisms composed of hyperadduction (reflected in lower and less variable *H_1_–H_2_* values and a tendency for higher CPP values) and associated increased laryngeal forces (negative skewing of the SPL distribution) play a prominent role in the singers' efforts to maintain singing and speaking vocal function in the presence of vocal fold lesions. Unfortunately, while this type of compensation may help to offset the detrimental impact of lesions on vocal function, in the long term, it is part of the pathophysiology of PVH that has the potential to cause further damage and deterioration of the voice (Hillman et al., 1989,^ 2020^).

Using the singing classifier to separately examine weeklong ambulatory recordings of singing and speech produced what potentially may be the most important findings of the current study. Based on post hoc testing of significant interaction effects for all three vocal doses, singers with phonotrauma had significantly higher vocal doses for speaking voice use than controls, with a tendency to have lower vocal doses than controls for singing. This simply means that singers with phonotrauma were spending more time talking than the healthy controls, but not significantly more time singing. Thus, our hypothesis that separating singing from speech will reveal differences between these two groups that have not been seen in previous studies is supported. Furthermore, these findings have potential implications in terms of the etiology of PVH in singers by suggesting that speaking voice use may play an important role.

Based on a consideration of all the results in the current study, we offer the following hypotheses about the etiology of PVH in singers. There is no doubt that singing is more vocally demanding than typical speaking as evidenced by higher and more variable levels of SPL and *f*_0_ and higher normalized vocal doses (Toles et al., 2020). However, increased use of speaking voice, in combination with the vocal demands of singing, has the potential to increase the risk of developing PVH, most probably because of the associated reduction in vocal recovery time. In addition, although speaking voice is considered less vocally demanding than singing, it is also associated with more abrupt/complete glottal closure (decreased *H_1_–H_2_* and increased CPP), which increases its potential to contribute to phonotrauma. We hypothesize that the increased use of speaking voice and the concomitant reduction in vocal recovery time lead to the onset of vocal fold tissue trauma. The onset of tissue trauma triggers a compensatory hyperfunctional response that includes hyperadduction of the vocal folds (reflected in decreased mean and variation for *H_1_–H_2_*) and increased laryngeal forces (reflected in negative skewing of the SPL distribution) in an effort to maintain vocal function. The compensatory response contributes to progressive development of established vocal fold lesions.

One obvious limitation of the current study is that patients were monitored after they were diagnosed with phonotrauma. It is impossible to identify which behaviors were present before developing vocal fold lesions and which occurred as a response to the presence of lesions. Furthermore, singers who have received a diagnosis of phonotrauma could potentially be limiting their singing voice use, out of recommendation from the treating physician or their own. Follow-up studies that monitor changes in vocal function as a result of successful treatment might be better able to address these issues. However, increased speaking voice use in singers is more likely to be a factor that is present prior to development of lesions, as some people are inherently more talkative and more prone to participate in social activities than others. We consider that personality factors might influence the likelihood of a person to exhibit increased speaking voice use. Patients with vocal fold nodules have been previously found to possess higher levels of personality traits related to extraversion (e.g., Social Potency), which have been hypothesized to predispose them to seek out social situations in which the voice is used to gain attention (Roy et al., 2000a, 2000b). These types of situations might include noisy social environments where the patient is not only speaking more frequently, but also louder than typical (Whittico et al., 2020). Though previous work related to the connection between personality and voice disorders has not included singers, it seems logical that singers with PVH would present with a similar personality profile as found by Roy et al. (2000a, 2000b), leading to increased speaking voice use. Further work that attempts to differentiate the personality traits of singers with PVH and healthy singers is warranted.

This is the first work to incorporate the use of the automatic singing classifier on ambulatory voice data to improve differentiation between patients and matched controls. The singing classifier demonstrated good accuracy during development and validation (Ortiz et al., 2019), so we have felt confident applying it to our data to objectively disaggregate the data into the two types of phonation. However, a major focus of our group is to continually improve methods for extracting phonatory measures from the neck surface acceleration signal. We have continued the task of further scrutinizing the performance of the singing classification system to ensure as few misclassifications as possible. After a subset of participants were removed from the data set, we felt even more confident in the results of the study. The disparity between misclassification rates in the validation study (Ortiz et al., 2019) and the current study was somewhat anticipated because the first study tested the classifier on data that was prelabeled as singing or speech by an expert listener, whereas the current study applied the singing classifier on unlabeled ambulatory voice recordings. Investigation into the misclassified frames among the participants that were removed from analysis revealed that much of the misclassified data seemed to include speech that was more “singsong-like.” The decision to use a binary classification system might be a limitation, considering that singing and speaking are on different ends of a continuum of phonation. There are clearly instances when individuals may produce phonation that combines characteristics of both singing and speech. Future work should consider modifying the singing classifier to allow for a nonbinary classification of phonation to probe further into the subtle differences along the continuum of phonation.

## Conclusions

Application of a singing classifier to weeklong ambulatory voice monitoring data revealed new differences between singers with PVH and matched controls. Consistent with previous studies of PVH, the pathophysiology of phonotrauma in singers is characterized by more abrupt/complete glottal closure (decreased mean and variation for *H_1_–H_2_*) and increased laryngeal forces (negatively skewed SPL distribution) during phonation. New results showed that singers spent more time speaking on a weekly basis (higher vocal doses), but not more time singing, when compared to vocally healthy matched controls.

Based on the results of this study, it is hypothesized that increased use of speaking voice, in combination with the vocal demands of singing, has the potential to increase the risk of developing PVH. This is probably due to a combination of reduced vocal recovery time and increased exposure to the more abrupt/complete glottal closure associated with the speaking voice use. It is further hypothesized that the onset of phonotrauma triggers compensation in the form of hyperadduction and increased laryngeal forces that may maintain vocal function but ultimately contributes progressive development of established vocal fold lesions.
